# Allele diversity for abiotic stress responsive candidate genes in chickpea reference set using gene based SNP markers

**DOI:** 10.3389/fpls.2014.00248

**Published:** 2014-06-05

**Authors:** Manish Roorkiwal, Spurthi N. Nayak, Mahendar Thudi, Hari D. Upadhyaya, Dominique Brunel, Pierre Mournet, Dominique This, Prakash C. Sharma, Rajeev K. Varshney

**Affiliations:** ^1^International Crops Research Institute for the Semi-Arid TropicsHyderabad, India; ^2^University School of Biotechnology, Guru Gobind Singh Indraprastha UniversityDelhi, India; ^3^Agronomy Department, University of FloridaGainesville, FL, USA; ^4^Etude de Polymorphisme des Génomes Végétaux, INRAEvry, France; ^5^UMR AGAP, CIRAD, Montpellier CedexFrance; ^6^UMR AGAP, Montpellier SupAgroMontpellier, France

**Keywords:** chickpea, abiotic stress, single nucleotide polymorphism, genetic diversity, candidate genes

## Abstract

Chickpea is an important food legume crop for the semi-arid regions, however, its productivity is adversely affected by various biotic and abiotic stresses. Identification of candidate genes associated with abiotic stress response will help breeding efforts aiming to enhance its productivity. With this objective, 10 abiotic stress responsive candidate genes were selected on the basis of prior knowledge of this complex trait. These 10 genes were subjected to allele specific sequencing across a chickpea reference set comprising 300 genotypes including 211 genotypes of chickpea mini core collection. A total of 1.3 Mbp sequence data were generated. Multiple sequence alignment (MSA) revealed 79 SNPs and 41 indels in nine genes while the *CAP2* gene was found to be conserved across all the genotypes. Among 10 candidate genes, the maximum number of SNPs (34) was observed in abscisic acid stress and ripening (*ASR*) gene including 22 transitions, 11 transversions and one tri-allelic SNP. Nucleotide diversity varied from 0.0004 to 0.0029 while polymorphism information content (PIC) values ranged from 0.01 (*AKIN* gene) to 0.43 (*CAP2* promoter). Haplotype analysis revealed that alleles were represented by more than two haplotype blocks, except alleles of the *CAP2* and sucrose synthase (*SuSy*) gene, where only one haplotype was identified. These genes can be used for association analysis and if validated, may be useful for enhancing abiotic stress, including drought tolerance, through molecular breeding.

## Introduction

Chickpea (*Cicer arietinum* L., 2*n* = 16), a self-pollinated, diploid annual species which ranks second worldwide as a food legume crop, is primarily a crop of developing countries contributing to a larger part of human food and animal feed in these areas. Chickpea is a major source of nutrients to a vegetarian diet as it contain 20–30% protein, ~40% carbohydrates and is also a good source of several minerals like calcium, magnesium, potassium, phosphorus, iron, zinc, and manganese. Global chickpea production is 11.6 million t from 12.3 million ha area with an average yield of less than one t/ha (FAO, 2012), much lower than its estimated potential of 6 t/ha under optimum growing conditions. Productivity of chickpea is adversely affected by several abiotic stresses of which drought, heat and cold are the major constraints affecting seed yield (Ruelland et al., 2002). Plant stress responses are generally controlled by a network of specialized genes through intricate regulation by specific transcription factors (Chen and Zhu, 2004). Application of available approaches to improve crop productivity under adverse environmental conditions requires a better understanding of the mechanisms involved during crop's response to abiotic stress. Genomic technologies and comparative genomics approaches that have emerged during the past decade can be exploited to identify some of the genes involved in drought tolerance mechanisms. Candidate genes for stress tolerance may be used in crop improvement programs directly (transgenic approach) or indirectly (through identification of linked SNPs) (Schena et al., 1995; Kudapa et al., 2013). The “chickpea mini core” comprising of 211 diverse genotypes (Upadhyaya and Ortiz, 2001) is a subset of the core collection (Upadhyaya et al., 2001) which represents the entire collection conserved in the ICRISAT Genebank. The reference set (Upadhyaya et al., 2008) includes four *C. reticulatum* genotypes and three *C. echinospermum* genotypes, but the majority (293 genotypes) is *C. arietinum* (Upadhyaya et al., 2006).

Although several genes have been found to be involved in abiotic stress tolerance in other crops, few studies have been carried out in chickpea. Candidate genes can be selected on the basis of prior knowledge from mutational analysis, biochemical pathways or linkage analysis of the trait of interest (Zhu et al., 2008). The candidate genes we selected were; Snf-1 related kinase (*AKIN*), amino-aldehyde dehydrogenase (*AMADH*), abscisic acid stress and ripening (*ASR*) gene, a homolog of the *DREB2A* gene, known as the *CAP2* gene, dehydrin (*DHN*), drought responsive element binding protein (*DREB*), *ERECTA*, Myb transcription factor (*MYB*), sucrose phosphate synthase (*SPS*), and sucrose synthase (*SuSy*).

The *AKIN* (*SNF1 related protein kinase*) gene belongs to the CDPK–SnRK superfamily, which serves as important regulators modulating fundamental metabolic pathways in response to nutritional and environmental stresses in plants (Halford and Hey, 2009). An *AMADH* gene in sorghum was found to be related to osmotic stress tolerance, dehydration and salt stress tolerance (Wood et al., 1996) and the activity of *AMADH* in response to stress caused by mechanical damage in pea seedlings was evaluated by Petrivalský et al. (2007). *AMADH* is expected to play a role in physiological processes and metabolic pathways controlling response to abiotic stresses by detoxification of toxic aminoaldehydes (Stiti et al., 2011). *ASR* gene is a stress-inducible gene that has been reported exclusively in plants and belongs to a small gene family characterized by the presence of an ABA/WDS domain. Members of the *ASR* gene family are induced by abscisic acid (ABA), various abiotic stresses including water stress and during the process of fruit ripening (Carrari et al., 2004). *ASR* genes in various species respond to different abiotic stress factors including drought, salt, cold and limited light (Joo et al., 2013). Over-expression of *ASR* in transgenic *Arabidopsis* was shown to increase tolerance to drought and salt and decrease sensitivity to exogenous ABA (Yang et al., 2005). Characterization of the *ASR* gene family in rice identified the *ASR3* gene as a candidate for association studies related to drought tolerance (Philippe et al., 2010). The potential importance of the *ASR1* gene in drought tolerance in common bean was reported by Cortés et al. (2012a) who found low nucleotide diversity suggestive of strong purifying selection, in wild and cultivated accessions.

*Dehydrins* (*DHNs*) are among the most commonly observed proteins induced by environmental stress associated with dehydration or low temperature (Hanin et al., 2011). The *DHN* proteins have been estimated to comprise up to 4% of the total seed protein, and are thought to be involved in protecting the embryo and other seed tissues from osmotic stresses associated with the low water content of the mature seed (Wise and Tunnacliffe, 2004). A positive correlation between accumulation of *DHN* proteins and tolerance to freezing, drought, and salinity has been shown (Close, 1996; Allagulova et al., 2003). Transgenic plants overexpressing *DHN* showed better growth and tolerance to drought and freezing stress compared to controls (Puhakainen et al., 2004). *DREB* are transcription factors that induce a set of abiotic stress-related genes and impart stress endurance to plants. *DREB*s belong to the *ERF* (ethylene responsive element binding factors) clade of the *APETALA2* (AP2) family are distinctive to plants. Transcription factors *DREB1A*/CBF3 and *DREB2A* were identified as cold and drought stress–responsive genes expressed in *Arabidopsis thaliana* (Sakuma et al., 2006). Constitutively activated *DREB2A* resulted in significant drought stress tolerance in transgenic *Arabidopsis* plants and expression analysis revealed that *DREB2A* transcriptionally regulates many water stress-inducible genes (Sakuma et al., 2006). In rice, expression of *OsDREB2A* was induced by dehydration and high-salt stresses (Matsukura et al., 2010; Mallikarjuna et al., 2011). Based on physiological studies in several crop species, the *DREB2A* transcription factor is one of the most promising candidate genes for drought tolerance. Low sequence diversity of *DREB2A* was found in five crop species studied; chickpea, common bean, rice, sorghum, and barley (Nayak et al., 2009) as well as in studies of wild and cultivated common bean (Cortés et al., 2012b).

The *ERECTA* gene codes for a protein kinase receptor which mediates plants' responses to disease, predation and stress. *ERECTA* is involved in leaf organogenesis and reduces the density of stomata on the leaf under-surface, thereby reducing the evapotranspiration. In *Arabidopsis*, the *ERECTA* gene has been shown to control organ growth and flower development by promoting cell proliferation (Shpak et al., 2004). The contribution of *ERECTA* gene toward water use efficiency was confirmed using complementation assays on wilting mutant *Arabidopsis* plants (Masle et al., 2005). The *ZmERECTA* genes from maize are patented by Pioneer Hi-Bred International, Inc., which were involved in improving plant growth, transpiration efficiency and drought tolerance in crop plants (www.freepatentsonline.com/y2008/0078004.html). The *Myb* transcription factor family constitutes the largest and diverse class of DNA-binding transcription factors in plants (Riechmann et al., 2000). The roles of *Myb* genes in response to biotic and abiotic stress have been studied in a number of plant species (Romero et al., 1998; Du et al., 2012; Volpe et al., 2013). *SuSy*, a glycosyltransferase, and *SPS* are key enzymes involved in sugar metabolism. Sucrose-synthase transcript and protein levels have been shown to be modulated by dehydration and rehydration (Kleines et al., 1999) and the *Arabidopsis AtSUS3* gene in particular was shown to be strongly induced by drought and mannitol, thus behaving as a marker of dehydrating tissues (Baud et al., 2004).

Genetic diversity, representing the overall genetic makeup of a species, serves as a basis for a population to adapt to changing environments (Ross-Ibarra et al., 2007). Single nucleotide polymorphisms (SNPs) have gained much popularity in assessing the diversity because of automation and abundance. Though biallelic SNPs are generally less informative than multi-allelic simple sequence repeats (SSRs), their sheer abundance makes the development of high density SNP genetic maps possible, providing the foundation for subsequent population-based genetic analysis (Rafalski, 2002). In addition, a SNP is of great importance if it affects gene function and the function of the gene in stress response is known/understood and the SNP is associated with differences in plant performance. Assessing genetic diversity for stress responsive candidate gene sequences leads to the identification of a specific allele of the particular gene in that species associated with performance in response to a corresponding abiotic stress. Such information can therefore be further used in breeding programs to develop better varieties using modern molecular breeding approaches like marker assisted recurrent selection (MARS) or gene pyramiding. Allelic diversity (richness), one of the most important and commonly used estimators of genetic diversity in populations, mainly depends on the effective population size and past evolutionary history (Petit et al., 1998). However, the number of alleles identified and their frequency distribution also depend on the genetic marker system used in these investigations. In the present study, the allelic diversity of candidate genes for abiotic stress tolerance was assessed in the chickpea reference set.

## Materials and methods

### Plant material and DNA extraction

Young leaf tissues of each accession of the reference set from the greenhouse grown plants were harvested and immediately stored in 96-well plate and the total genomic DNA of all the genotypes was isolated using high-throughput mini- DNA extraction method (Cuc et al., 2008). The quality and quantity of extracted DNA was checked on 0.8% agarose gel. The DNA was normalized to 20 ng/μl concentration for further use.

### Identification of abiotic stress responsive genes and primer designing

A set of 10 abiotic stress responsive genes conferring abiotic stress tolerance in model plants (*Arabidopsis* and Rice) and other crop species (*Glycine max* and *Medicago* spp.) were chosen based on available literature (Table 1). Different approaches were used for primer designing based on availability of gene sequence information in chickpea. In the first approach, heterologous primers were designed for *ASR, SuSy*, and *SPS* genes from corresponding *Medicago* sequences. The *ERECTA* gene in chickpea was isolated using consensus/degenerate primers designed at INRA, EPGV, France. In the second approach sequence-specific primers were designed, where in chickpea homologs of genes were isolated using chickpea ESTs developed for abiotic stress (Varshney et al., 2009) and available in NCBI EST database (DbEST- http://www.ncbi.nlm.nih.gov/dbEST/) (Roorkiwal and Sharma, 2012). The details of primers used in isolation of abiotic stress responsive candidate genes in chickpea are given in Table 1.

**Table 1:** **List of abiotic stress responsive genes and respective primers used for PCR amplification**.

**Gene**	**Putative function**	**Source sequence**	**GenBank/TC ID**	**Primer sequences (5′–3′)**
SNF-1 related protein kinase (*AKIN*)	Response to nutritional and environmental stresses in plants	Chickpea ESTs	–	F: GTG GTT CAG GTG CAG ACT TG
				R: TCA GAA AGT GCC CAT CAC GC
Aminoaldehyde dehydrogenase (*AMADH*)	Osmotic stress, dehydration and salt stress tolerance	Chickpea ESTs	–	F: TTG GAA GAA GGT TGC AGG CTA G
				R: CCC ATT CTC CCA GTT CAC GG
Abscisic acid stress and ripening (*ASR*)	Tolerance to drought and salt stresses	*Medicago*	AC152054	F: GGG AAC TAA TCC TTT CCA AAC A
				R: CTG CAG CAC CTA ACT CAC CA
*CAP2* gene (*DREB2A*)	Regulates expression of water stress-inducible genes	Chickpea	DQ321719	F: CGG CTT CCC TTC ATT CGA TCC A
				R: AGG CAC AAC ACA AGA ATC CA
*CAP2* promoter	Induce a set of abiotic stress-related genes	Chickpea	–	F: TGT GCT TCA AGT TGC ACT CC
				R: CGG GGT CCT TAT ATA CTG CAG A
Dehydrin (*DHN*)	Induced by environmental stress, dehydration or low temperature	Chickpea ESTs	–	F: AAA GTG GTG TTG GGA TGA CC
				R: TCC TCT CTC CCG AAT TCT TG
Dehydration responsive element binding (*DREB1*)	Induced by dehydration and high-salt stresses	Chickpea ESTs	–	F: CTT CAT TCG ATC CAG ATT CGG
				R: AAC GCG AGT TTT CAG GCC CT
*ERECTA* (fragment 7F-5R)	Mediates plants' responses to disease and stress	Degenerate	–	F: GTG TAC AAA CCT TAA CAG CC
				R: CCA GTT AAT TCG TTG TTT TC
*ERECTA* (fragment 8F-8R)	Mediates plants' responses to disease and stress	Degenerate	–	F: GGT CAG CTA CAG AAC ATA GCA
				R: TCC ATT TTC CAT GTA GTC ATA A
*Myb* transcription factor	Response to biotic and abiotic stresses	Chickpea ESTs	–	F: ATG CTA CTG CTG CCT ACA AG
				R: ACC GCA GTA CAC TCC AAG AG
Sucrose synthase (*SuSy*)	Sugar metabolism pathway	*Medicago*	TC95820	F: GAT ACT GGC GGA CAG GTT GT
				R: CAT CCT TTG CTA GGG GAA CA
Sucrose phosphate synthase (*SPS*)	Induced by drought and mannitol	*Medicago*	BQ137986	F: TTT GGT CCA CGC GAT AAA TA
				R: TGA ATT GAT ATC CTC CCA AGA

### Polymerase chain reaction (PCR) and sequencing of amplicons

In order to amplify these candidate genes and confirm their presence, a pilot experiment was set to sequence amplicons from eight diverse genotypes of chickpea consisting of Annigeri, ICCV 2, ICC 4958, ICC 1882, ICC 283, ICC 8261, ICC 4411, and ICC 10029. PCR was set up with 20 μl reaction mixture comprising 5 ng of template DNA, 5 picomoles each of forward and reverse primers, 2 mM dNTP, 20 mM MgCl_2_, 1X PCR buffer (AmpliTaq Gold) and 0.25 U of Taq polymerase (Ampli *Taq* Gold). PCR cycles comprising of denaturation of 94°C for 5 min, followed by 40 cycles of 94°C for 30 s annealing at temperature specific for each target gene for 40 s and 72°C for 1 min 30 s and a final extension was carried out at 72°C for 20 min. The amplified product (about 2 μl) was loaded on 1.2% agarose. The remaining PCR amplicons were purified using 1 unit of Exonuclease I and 1 unit of shrimp alkaline phosphatase (SAP) per 5 μ l of PCR product. The Exo/SAP added PCR products were incubated for 45 min at 37°C followed by denaturing at 80°C for 15 min in the thermal cycler for deactivating unused exonuclease enzyme. The Exo/SAP treated amplicons were mixed with 1 μ l of BigDye Terminator V3.1 (Applied Biosystems, California, USA), 2 μ l of 5X sequencing dilution buffer and 3.2 μ M of primer (forward and reverse, separately) and the volume was made to 10 μ l by adding water. The sequencing PCR profile included an initial denaturation of 96°C for 30 s, followed by 60 cycles of 96°C for 10 s, 50°C for 5 s, and 60°C for 4 min. The PCR products were stored at 4°C until further use. Before sequencing, the PCR products were treated with 2.5 μ l of 125 mM EDTA and 25 μ l of absolute ethanol and incubated for 15 min at room temperature to precipitate the DNA. The plate containing the PCR product was centrifuged at 4000 rpm for 30 min at 4°C. The ethanol/ EDTA mix was poured off by inverting the plate, without losing the pellet. To each well, 60 μ l of 70% ethanol was added and again spun at 4000 rpm for 20 min at 4°C. The ethanol was poured off as earlier. The plate was air-dried and 10 μ l of HiDi formamide (Applied Biosystems, California, USA) was added and the products were denatured (94°C for 5 min, then immediately cooled to 4°C for 5 min) and sequenced using an ABI3700/ABI3130 automated sequencer (Applied Biosystems, California, USA).

### Allele sequencing and SNP detection

For allele sequencing, of candidate genes across the 300 genotypes of the reference set, PCR and purification were carried out as described above. Sequencing was carried out at MACROGEN, Korea using BigDye terminator cycle sequencing chemistry. Raw sequences were used to obtain contigs by assembling the forward and reverse sequences of each genotype using DNA Baser V 2.9 tool and gene identities were confirmed using BLAST (blastn and blastx). The sequences of each candidate gene were aligned using CLUSTALW (http://www.ebi.ac.uk/Tools/clustalw2/index.html). Multiple sequence alignment (MSA) files and fasta files were further used for identifying equence related parameters such as number of genotypes sequenced; length of sequences; number of indels; indel frequency; number of SNPs and their types (transition or transversion); SNP frequency; nucleotide and haplotype diversity and polymorphic information content (PIC) of SNPs and haplotypes using an in-house tool developed at ICRISAT called “DIVersity ESTimator” module (DIVEST) (Jayashree et al., 2009). Further, in order to identify if any of the haplotypes could be associated with the country of origin of the genotypes under study, NETWORK programme version 4.516 was used to determine haplotype networks for each candidate gene studied.

## Results

### Isolation and sequence analysis of abiotic stress responsive candidate genes

An *AKIN* homolog was amplified using the gene specific primer pair designed considering unigene sequence showing match with *Arabidopsis AKIN* (SNF-1 related protein kinase). The approximate amplicon size of *AKIN* was ~800 bp. Amplification of an *AMADH* homolog yielded a product of ~900 bp. The ABA stress and ripening (*ASR*) gene was isolated using the heterologous primers derived from *Medicago* sequence AC152054. A single amplicon of 700 bp was obtained for the chickpea genotypes used. A *DREB2A* homolog (also known as *CAP2* gene) and its promoter (*CAP2* promoter) were amplified using a primer pair as described by Nayak et al. (2009). The approximate amplicon size of the *CAP2* gene was 1000 bp while the *CAP2* promoter was ~700 bp. A dehydrin homolog of chickpea was amplified using a primer pair designed for known dehydrin gene using chickpea unigene. The approximate amplicon size of dehydrin gene was ~380 bp. A *DREB1* (Dehydration response element binding) homolog in chickpea was also amplified using a primer pair designed using unigene showing match against *DREB1* gene. The approximate amplicon size of the *DREB1* gene was ~800 bp. About 4300 bp long ERECTA gene fragments were isolated from eight chickpea genotypes using consensus primers. An ~350 bp long *MYB* gene was amplified using unigene sequence having match against *Glycine max* Myb transcription factor. For isolating the *SuSy* gene in chickpea, heterologous primers were designed from *Medicago* sequences TC95820 (homolog to *SUS2* Pea) and AJ131964 (*Medicago truncatula SUS1* gene). An ~1500 bp amplicon was obtained for TC95820- derived sequences, while a 900 bp amplicon was obtained with AJ131964- derived sequences. Heterologous primers designed using *Medicago* sequence BQ137986 and CB893717 were used to isolate *SPS* in chickpea. Amplification across eight genotypes in chickpea yielded products of 400 bp in both cases (Table 2).

**Table 2:** **Summary of abiotic stress responsive candidate genes showing match with previously reported accession/gene in other crop species**.

**Gene**	**Sequence length (bp)**	**Sequence similarity result**	***e*-value**
SNF-1 related protein kinase (*AKIN*)	772	SNF1-related protein kinase catalytic subunit alpha KIN10 [*Arabidopsis thaliana*] AKIN10	6.00E-41
Aminoaldehyde dehydrogenase (*AMADH*)	932	Betaine aldehyde dehydrogenase 1 [*Arabidopsis thaliana*]	2.00E-36
Abscisic acid stress and ripening (*ASR*)	680	(1) TC10668 similar to *ASR* protein homolog	2.80E-18
		(2) *Medicago truncatula* clone (AC126014.6)	3.00E-29
		(3) *Prunus armeniaca* (apricot) *ASR* (U93164.1)	0.003
*CAP2* gene (*DREB2A*)	1000	DQ321719 (*CAP2* gene *Cicer arietinum*)	0.00
*CAP2* promoter	700	–	–
Dehydrin (*DHN*)	381	Dehydrin 1 [*Cicer pinnatifidum*]	2.00E-04
Dehydration responsive element binding (*DREB1*)	776	Dehydration responsive element binding protein [*Cicer arietinum*]	2.00E-09
*ERECTA*	4300	LRR receptor-like serine/threonine-protein kinase ERECTA [*Medicago truncatula*]	
Myb transcription factor (*MYB*)	335	(1) MYB transcription factor MYB93 [*Glycine max*]	2.00E-26
		(2) Myb-like transcription factor family protein [*Arabidopsis thaliana*]	0.00
Sucrose phosphate synthase (*SPS*)	400	(1) *M. truncatula* (BQ137986) *SPS* like protein	7.90E-60
		(2) TC103232 homolog to *Medicago sativa* SPS (Q9AXK3)	9.60E-21
Sucrose synthase (*SuSy*)	900	(1) *M.truncatula SusS1* gene (AJ131964)	2.00E-20
		(2) *Lotus japonicus* genomic DNA clone (AP009336.1)	3.00E-18
		(3) *Vigna radiata* mRNA for *SUSY*(D10266.1)	3.00E-06

### Sequence diversity analysis of candidate genes

Forward and reverse sequences for all 10 abiotic stress responsive candidate genes and the *CAP2* gene promoter, were used for contig construction. The number of genotypes for which good quality sequences were obtained varied from 79 (*ERECTA* fragment obtained from 7f-5r primer pairs) to 236 genotypes (*SPS* gene), out of the 300 genotypes. Diversity analysis of the candidate genes using the DIVersity ESTimator (DIVEST) tool is presented in Table 3.

**Table 3:** **Estimation of sequence diversity in chickpea reference set/mini core collection using 10 abiotic stress responsive genes**.

**Candidate gene**	***AKIN*^#^**	***AMADH*^#^**	***ASR***	***CAP2***	***CAP2* promoter**	***DHN*^#^**	***DREB1*^#^**	***ERECTA* _7f_5r**	***ERECTA* _8f_8r**	***Myb^#^***	***SPS***	***SuSy***
Genotypes with successful sequences	208	209	193	227	137	198	191	79	147	200	236	230
Sequence length (bp)	772	932	621	367	629	381	776	921	1189	335	312	884
No. of Indels	2	3	2	0	0	7	23	1	0	2	1	0
Indel frequency	1/386.00	1/310.67	1/310.60	0	0	1/54.43	1/33.74	1/921.00	0	1/167.50	1/312.00	0
No. of SNPs	2	13	34^*^	0	1	7	14	13	20	6	3	1
Transition	2	6	22	0	0	5	8	9	10	1	2	1
Transversion	0	7	11	0	1	2	6	4	10	5	1	0
SNP frequency	1/386.00	1/71.69	1/18.26	0	1/629.00	1/54.43	1/55.43	1/70.86	1/69.46	1/55.83	1/104.00	1/884.00
Nucleotide diversity (Pi)	0.0004	0.002	0.0014	0	0	0.0022	0.0011	0.0029	0.0029	0.002	0.0011	0.0012
Average PIC of SNP	0.01	0.04	0.1	0	0.43	0.17	0.14	0.27	0.1	0.04	0.01	0.01
No. of haplotypes	3	9	4	1	2	6	33	4	3	6	4	1
Haplotype diversity	0.019	0.326	0.833	0	0.438	0.426	0.879	0.372	0.324	0.256	0.034	0.035
PIC of haplotypes	0.019	0.324	0.829	0	0.436	0.424	0.874	0.367	0.322	0.255	0.034	0.033

The sequence diversity was calculated using DIVEST tool (http://hpc.icrisat.cgiar.org/Pise/5.a/statistics_calculation/SNP_diversity_estimator.html) AKIN, SNF1 related protein kinase; AMADH, Aminoaldehyde dehydrogenase; ASR, Abscisic acid stress and ripening gene; DHN, Dehydrin; DREB1, Dehydration responsive element binding protein; Myb, Myb transcription factor; SPS, Sucrose synthase (SuSy) and sucrose phosphate synthase;

# Gene was sequenced across 211 genotypes of chickpea mini core collection;

* One SNP is tri-allelic.

**Figure 1:**
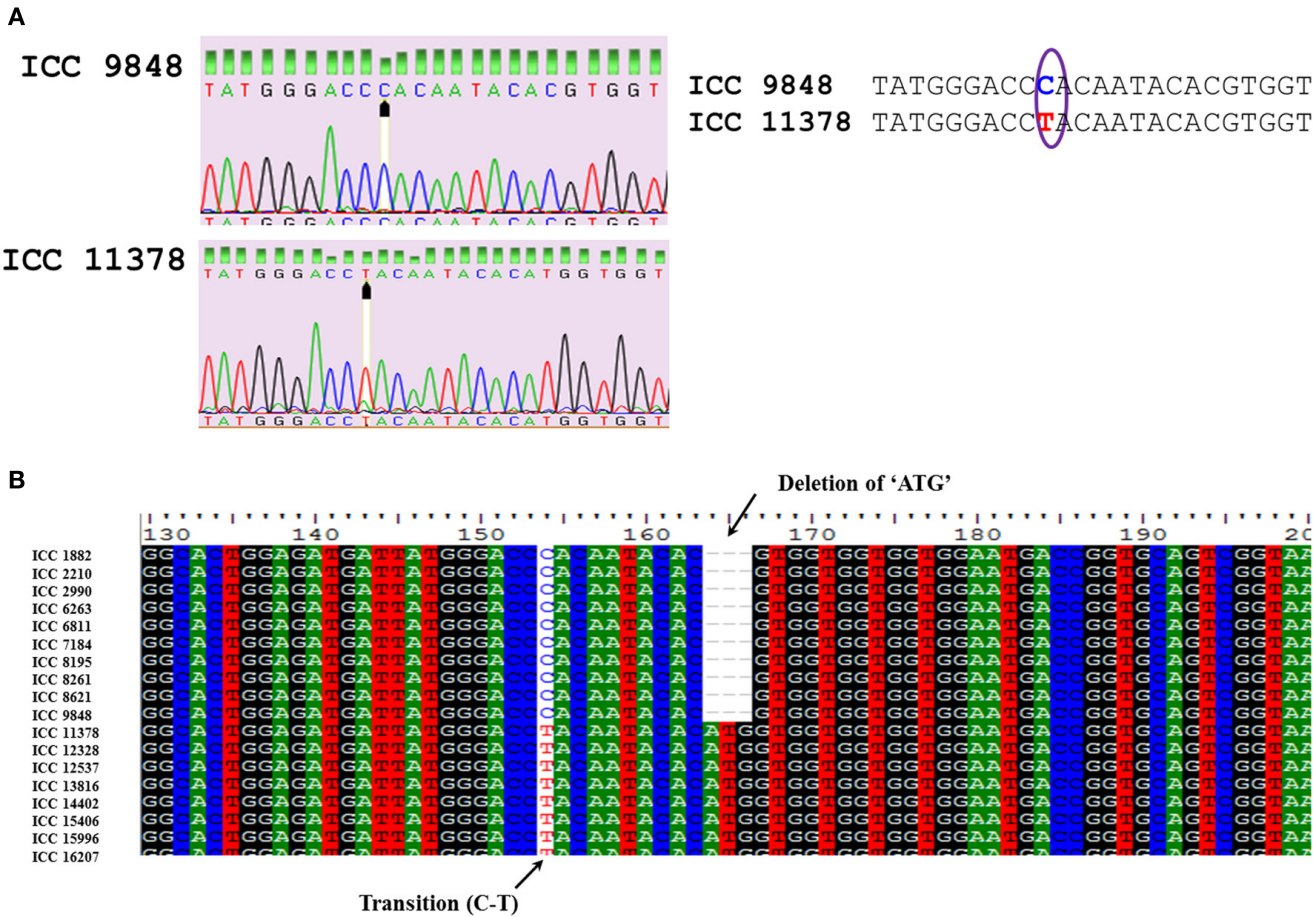
**(A)** Comparison of sequence quality to confirm the true SNP using peak quality. The presence of SNP in *DHN* gene using sequence chromatogram is highlighted. **(B)**. Alignment of nucleotide sequences encoding *DHN* across various chickpea genotypes.

SNPs were manually inspected for possible sequencing errors and only those SNPs with clear peaks were considered further (Figure 1A). Sequences for each gene were aligned using CLUSTALW and positions of SNPs were identified (Figure 1B). The highest number of SNPs (34) was obtained for the *ASR* gene, amongst which 22 were transitions, 11 were transversions and one was tri-allelic. Apart from SNPs, two indels were also detected. The *CAP2* gene was found to be conserved across all 227 genotypes with no SNPs and indels. In the case of *CAP2* promoter, one SNP was found (which was the same observed when eight chickpea genotypes were sequenced as a pilot experiment). For the *ERECTA* gene, two fragments obtained from 7f-5r and 8f-8r primer pairs were sequenced. In total, 13 SNPs (9 transitions and 4 transversions) and one indel were obtained for *ERECTA* 7f-5r fragments while 20 SNPs (10 transitions and 10 transversions) were observed for *ERECTA* 8f-8r gene fragments. One indel and 3 SNPs were observed across *SPS* gene sequences. The *AKIN* gene showed the presence of two SNPs and two indels. A total of 13 SNPs (6 transitions and 7 transversions) and 3 indels were identified in the *AMADH* gene, while in the *DHN* gene 7 SNPs (five transitions and two transversions) were identified among 198 sequences analyzed. For the *MYB* gene only 6 SNPs (one transition and five transversions) and 2 indels were found in 200 *Myb* sequences under study. No nucleotide diversity was observed for the *CAP2* gene and promoter while in the case of *AKIN* it was 0.0004 and 0.0029 for both *ERECTA* fragments. The average polymorphic information content (PIC) value of SNPs ranged from 0 (*CAP2* gene) to 0.43 (*CAP2* promoter). Haplotype diversity ranged from 0.019 (*AKIN*) to 0.879 (*DREB1*). Average (PIC) of haplotypes values ranged from 0.019 (*AKIN*) to 0.874 (*DREB1*) (Table 3).

### Haplotype networks for candidate genes

**Figure 2:**
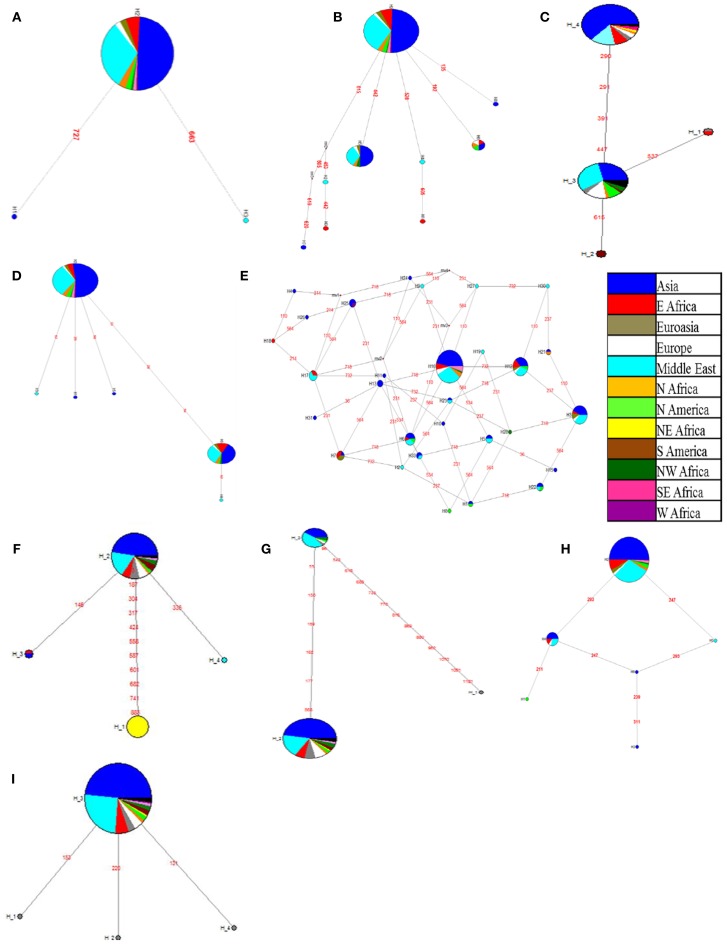
**Haplotype network of candidate genes developed based on country of origin of genotypes of the chickpea reference set. (A)** *AKIN* gene; **(B)** *AMADH* gene; **(C)** *ASR* gene; **(D)** *DHN* gene; **(E)** *DREB1* gene; **(F)** *ERECTA* (*7f-5r*) gene; **(G)** *ERECTA* (*8f-8r*) gene; **(H)** *MYB* gene; **(I)** *SPS* gene; Each circle represents a haplotype and is labeled accordingly. Colors in the circles represent the countries of origin of chickpea genotypes. Circle size is in proportion to frequency (the larger the circle the more genotypes in the haplotype). Numbers in red represent the position of mutations separating the haplotypes.

Based on the sequence information, haplotype networks were drawn using the NETWORK program. The network figures show the number of haplotypes observed for each gene and the SNP position which separates one haplotype from the other. Network diagrams can be drawn only with the presence of more than two haplotype blocks. Haplotype frequency is depicted by circles, for example, the larger the haplotype circle, more genotypes are represented by that haplotype. The color code is given as per the country of origin of the genotypes (Figures 2A–I). *CAP2* and *SuSy* gene represented only one haplotype with all the genotypes sequenced while the *CAP2* promoter had only one SNP, forming two haplotype blocks. Hence haplotype network graphs could not be drawn for *CAP2* gene, its promoter and *SuSy* gene. The network analysis showed a linear relationship between haplotypes for most of the genes except for transcription factors *DREB1* and *Myb*, which showed network relationships between larger numbers of haplotypes.

In this study, although we could find more than two haplotype blocks in some of the candidate genes like *AKIN, AMADH, ASR, DHN, DREB, MYB, SPS, ERECTA* (7f-5r), and *ERECTA* (8f-8r), there was no clear distinction between the origin of the genotypes and the haplotype information. Haplotype network analysis for the *AKIN* gene reported three haplotypes, including one major (H2) and two minor haplotypes (H1 and H3) (Figure 2A). The *AMADH* gene showed the presence of nine haplotypes across the reference set of which, one major haplotype (H9) is connected to eight other haplotypes (Figure 2B). There were three minor haplotypes (H1, H2, and H4) derived from a major haplotype (H3) as observed in *ASR* haplotype networks with SNPs ranging from one to four (Figure 2C). *DHN* gene haplotype network indicated the presence of six haplotypes, of which one major haplotype (H2) was connected to three minor haplotypes (H1, H3, and H5) with one SNP and another haplotype (H6) with three SNPs which was further connected to one minor haplotype (H4) with one SNP (Figure 2D). The *DREB1* gene exhibits a complex haplotype network owing to the presence of 33 different haplotypes, which were connected to each other with 1–4 SNPs (Figure 2E). Three major haplotypes (H3, H12, and H16) covers 17, 19, and 62 individuals respectively (Figure 2E). Similarly, in *ERECTA*- 7f-5r gene fragment, one major haplotype (H1) defined by 10 SNPs and two minor haplotypes (H3 and H4) defined by single SNP were derived from major haplotype H2 (Figure 2F). In the case of the other *ERECTA* fragment (8f-8r) two haplotypes (H1 and H2) derived from H3 with 6 and 13 SNPs respectively (Figure 2G). Haplotype network of *Myb* gene showed the presence of six haplotypes, of which two major haplotypes (H2 and H4) are connected to four minor haplotypes with 1–2 SNPs (Figure 2H). SPS gene haplotype network showed presence of three minor haplotypes (H1, H2, and H4) derived from H3 with single nucleotide variation (Figure 2I). Accessions representing each haplotype were color coded according to their country of origin. In the present study, accessions in the major haplotypes were coming from Asia and Middle East in all the genes. The haplotype for ERECTA 7f-5r is unique to NE Africa. The network analysis showed linear relation between haplotypes in most of the genes except for *DREB1* and *Myb*, which are transcription factors. It is also interesting to note that these are the transcription factors which regulate many downstream genes in plant system.

## Discussion

The present study was initiated with the objective of the identification of favorable alleles in abiotic stress responsive genes in the chickpea reference set. These gene-based SNPs may be used to identify the suitable allele of a gene that enable the plant to survive in a stress environment. Due to lack of genome sequence information of the chickpea genome until recently (Varshney et al., 2013), identification of genes responsible for complex traits like drought tolerance was a daunting task at the time of initiation of this study. Identification of candidate genes responsible for drought tolerance was a part of an international collaborative project funded by the Generation Challenge Programme (GCP) entitled “Allelic Diversity at Orthologous Candidate genes (ADOC) in seven GCP crops”- one among them was chickpea. An extensive literature survey was carried out to identify possible candidate genes responsible for abiotic stress tolerance, which might have a consensus role in abiotic stress tolerance mechanism in model crops and other legume crops.

Most of the genes analyzed here, have not been previously studied in chickpea. Therefore, systematic efforts by using comparative genomics and bioinformatics approaches were made to determine the corresponding gene sequences in chickpea. For instance, a *DREB* homolog of chickpea was isolated by using sequence information available from chickpea. As *Medicago truncatula* is the known taxonomic ally of chickpea, the genomic information about *Medicago* was searched from different databases including NCBI, TIGR, and *Medicago* sequence repository (www.medicago.org). Putative candidate genes in chickpea namely *ASR, SuSy* and *SPS* were isolated using respective sequence information obtained from the *Medicago* candidate gene sequences. In addition, the remaining abiotic stress responsive genes (*AKIN, AMADH, DHN*, and *MYB*) were identified using a sequence similarity approach against the homolog genes present in model crops like *Arabidopsis* and *Medicago*. A large body of evidence demonstrated that the Snf1-related protein kinases (*AKIN*) serve as important regulators modulating fundamental metabolic pathways in response to nutritional and environmental stresses in yeast and mammalian cells (Hardie, 2007). To identify the *AKIN* homolog, chickpea ESTs were used for designing the primers for PCR amplification in eight chickpea genotypes based on sequence similarity with *Arabidopsis thaliana* (Table 2). Researchers have isolated the *AKIN* homolog in various plant species including *Arabidopsis*, wheat, rice, potato and tobacco and have established their role in abiotic stress response (Coello et al., 2012). The *AKIN* gene encodes two types of domains, catalytic kinase (highly conserved) domain and regulatory domain (highly divergent). In the present study, the *AKIN* gene was found to be mostly conserved except two unique alleles each reported in specific genotype, which indicates that in the present study we were able to amplify the conserved part of *AKIN* gene, i.e., catalytic kinase. Researchers can target the divergent regulatory domain to identify the SNPs actively involved in abiotic stress response. Similarly, a protective/curative role of the *AMADH* gene in response to stress events caused by mechanical injury was reported by Petrivalský et al. (2007) in pea seedlings. Since *AMADHs* works on degradation of reactive metabolites that show considerable toxicity, this enzyme was thought to serve as a detoxification enzyme. An *AMADH* homolog was amplified using primers designed from chickpea ESTs and BLASTN analysis confirmed its presence (Table 3). Over expression of the *AMADH* genes from *Arabidopsis* have been shown to affect stress responses (Missihoun et al., 2011). Based on various functional and characterization studies of the *AMADH* gene in rice, *Arabidopsis* and other crop species (Skibbe et al., 2002; Tsuji et al., 2003) makes this gene a suitable candidate for studying its similar role in chickpea. In our study, *AMADH* showed the second highest number of SNPs (13) across the chickpea mini core collection.

Expression of the *ASR* gene is regulated by water stress, salt stress and plant hormone ABA. Over-expression of the *ASR* gene in transgenic plants is known to induce water- and salt- stress tolerance (Kalifa et al., 2004). Although *ASR* gene function is not published in the case of *Medicago, ASR*-like sequences that were similar to some of the reported ASR sequences in other crops were used to design primers and amplified in chickpea. The sequence diversity across chickpea genotypes (193 sequences) showed 34 SNPs and two indels, highest among the candidate genes studied in the present study. The nucleotide diversity was found to be 0.0014 while haplotype diversity was 0.833. Cortés et al. (2012b) also analyzed the diversity of two *ASR* genes in a set of wild and cultivated beans and found two contrasting diversity patterns, most particularly for wild beans. A similar study in rice was carried out, where the polymorphism of four members of the *ASR* gene family was studied in a worldwide collection of 204 accessions of *Oryza sativa* and 14 accessions of wild relatives (*O. rufipogon* and *O. nivara*). This study provided a thorough description of the organization of the *ASR* family, and the nucleotide and haplotype diversity of four *ASR* genes in *O. sativa* (Philippe et al., 2010).

The chickpea *CAP2* gene (a homolog of *DREB2A*) and its promoter, known to enhance tolerance to dehydration and salt stress, were isolated, characterized and expression studies were carried out in transgenic tobacco (Shukla et al., 2006). The sequence information was used to design nested primers in order to isolate the full-length *CAP2* gene during the present study. The study also showed extreme conservation of the *AP2* domain of the *DREB2* genes across five species studied (Nayak et al., 2009). *DREB* transcription factors bind to the dehydration responsive element (DRE) of the genes at the promoter region and regulate the expression of downstream genes. The DRE containing core sequence A/GCCGAC was identified as a cis-acting promoter element, which regulates gene expression in response to drought, high salinity and cold stresses in *Arabidopsis* (Yamaguchi-Shinozaki and Shinozaki, 1994). The *CAP2* gene and its promoter were sequenced in 300 diverse chickpea genotypes. The occurrence of a SNP within a regulatory region, accounting for the loss of function of a seed shattering gene has been already shown in rice, which indicates that single sequence variants can cause major effects on the function of gene(s) (Konishi et al., 2006). Conservation of the *AP2* domain of the *DREB2A* gene was observed, not only within chickpea sequences, but also across other crop species; common bean, rice, sorghum and barley (Nayak et al., 2009). *DREB2A* diversity analysis in common bean (Cortés et al., 2012a) revealed a very high diversity level compared to *DREB2B* in these other species, indicative of adaptive selection and population expansion.

The *DHNs* are one of the several proteins that have been specifically associated with qualitative and quantitative changes in cold hardiness (Close, 1996). *Arabidopsis* plants engineered for *DHN* over-expression, showed improved survival when exposed to low temperature (Puhakainen et al., 2004). Similarly, transgenic tobaccos with increased level of expression of a citrus dehydrin protein have shown tolerance to low temperature (Hara et al., 2003) making *DHN* a suitable candidate gene for study in chickpea. Researchers have distinguished five different *DHN* genes *in silico*, which could be grouped into two types-*K2* and *SKn*. Three of the dehydrin genes reported several sequence variants which differ by multiple or single amino acid substitutions (Velasco-Conde et al., 2012). The role of *ERECTA* genes in drought tolerance pertains to their involvement in stomatal density and evapotranspiration (Shpak et al., 2004; Masle et al., 2005). Two fragments of *ERECTA* genes were isolated in the present study. In chickpea, a total of 33 SNPs (13 from fragment obtained from *ERECTA*-7f-5r and 20 from fragment obtained from *ERECTA*-8f-8r) making 7 haplotypes (4 in *ERECTA*-7f-5r and 3 in *ERECTA*-8f-8r) were observed. Nucleotide diversity was found to be 0.0029 which was high compared to all other candidate genes under study. The sequence diversity studies across the reference set of chickpea, provides the insights regarding existing haplotypes, which could be involved in drought tolerance mechanism. The role of plant Myb-proteins has been well characterized by using different genetic approaches. In most of the cases, the *Myb* domain binds to a specific DNA sequence (C/TAACG/TG) to facilitate transcriptional activation (Biedenkapp et al., 1988). A rice R2R3-type MYB transcription factor gene, *JAmyb*, whose overexpression causes tolerance to high salinity has been identified (Yokotani et al., 2013).

The *SuSy* and *SPS* genes encode for the enzymes involved in sugar metabolism and are known to be up-regulated in dehydration stress. The *SuSy* gene in chickpea is also associated with increased seed size (Kumar and Turner, 2009). A partial *SuSy* gene was isolated here, and sequencing discovered only 1 SNP across the chickpea reference set. The *SuSy* gene is a candidate gene for drought tolerance in many plant species (Gonzalez et al., 1995; Baud et al., 2004), and the *SPS* gene was found to be involved with drought tolerance in maize (Abdel-latif, 2007) and wheat (Fresneau et al., 2007). An *SPS* homolog was identified in chickpea in the present study. Diversity analysis of this gene on the reference set of chickpea showed the presence of three SNPs and one indel represented as four haplotypes across 235 chickpea genotypes. This observation indicates the conservation of this gene across chickpea genotypes. Studies on sequence diversity on the *SPS* gene are limited to date. Sequence diversity of an *SPS* gene was studied for two cultivars of sugarcane and 10 SNPs were identified in a 400 bp sequenced region. These SNPs were screened on a mapping population derived from the two cultivars. The SNP frequency did not vary in the two bulked DNA samples, suggesting that SNPs from this *SPS* gene family are not associated with variation in sucrose content. Estimation of genetic diversity serves many purposes concerning breeder's interest, like identification of distinct genetic groups for retention in germplasm, identification of genes responsible for phenotypic variation accrued during domestication (Ross-Ibarra et al., 2007) and inference of crop evolution. Allelic diversity studied through NETWORK indicated the distribution of different alleles across the globe based on the origin of the accessions. For some genes (ex: ERECTA 7F-5r), haplotypes identified were coming from particular geographic area (ex: H1 from NE Africa). Such haplotypes indicate a historical constraint as a result of selection, domestication or adaptation. In rice, a haplotype study of three genes revealed the difference in domestication pattern of cultivated and wild rice cultivars (Londo et al., 2006; Kovach et al., 2007). In the present study, linear haplotype networks were found in all genes except for transcription factors *DREB1* and *Myb*. Diversity of transcription factors at a sequence and functional level may affect downstream genes and their expression. Knowledge about genetic diversity and relationships within the diverse germplasm is also useful for breeders as it facilitates their decisions on the selection of the parents for hybridization when widening the genetic basis of breeding programs. Molecular variation in the germplasm can help in the selection of superior genotypes for the generation of new varieties for several agronomic traits. A total of 114 SNPs and 41 indels have been identified in these abiotic stress responsive genes across the chickpea reference set. These SNPs and indels were used for diversity estimation using DIVersity ESTimator (DIVEST). Among the 114 SNPs detected, 66 SNPs regions were transitions, whereas the other 49 were transversions, and one SNP was reported tri-allelic. The nucleotide diversity across the chickpea mini core collection ranged from 0.0004 to 0.0022 with overall mean diversity of 0.0015. The possibilities of association mapping can be explored further by linking sequence diversity with the phenotype diversity in order to identify favorable alleles or haplotypes conferring drought tolerance in chickpea.

### Conflict of interest statement

The authors declare that the research was conducted in the absence of any commercial or financial relationships that could be construed as a potential conflict of interest.
